# Dysregulated hepatic lipid metabolism and gut microbiota associated with early-stage NAFLD in ASPP2-deficiency mice

**DOI:** 10.3389/fimmu.2022.974872

**Published:** 2022-11-18

**Authors:** Fang Xie, Hang-fei Xu, Jing Zhang, Xiao-ni Liu, Bu-xin Kou, Meng-yin Cai, Jing Wu, Jin-ling Dong, Qing-hua Meng, Yi Wang, Dexi Chen, Yang Zhang

**Affiliations:** ^1^ Beijing Institute of Hepatology, Beijing Youan Hospital, Capital Medical University, Beijing, China; ^2^ Beijing Engineering Research Center for Precision Medicine and Transformation of Hepatitis and Liver Cancer, Beijing Institute of Hepatology, Beijing, China; ^3^ Department of Hepatology, Beijing Youan Hospital, Capital Medical University, Beijing, China; ^4^ State Key Laboratory of Proteomics, Beijing Proteome Research Center, National Center for Protein Sciences (Beijing), Beijing Institute of Lifeomics, Beijing, China

**Keywords:** ASPP2, NAFLD, fatty acid, lipid metabolism, gut microbiota

## Abstract

**Background:**

Growing evidence indicates that lipid metabolism disorders and gut microbiota dysbiosis were related to the progression of non-alcoholic fatty liver disease (NAFLD). Apoptosis-stimulating p53 protein 2 (ASPP2) has been reported to protect against hepatocyte injury by regulating the lipid metabolism, but the mechanisms remain largely unknown. In this study, we investigate the effect of ASPP2 deficiency on NAFLD, lipid metabolism and gut microbiota using ASPP2 globally heterozygous knockout (ASPP2^+/-^) mice.

**Methods:**

ASPP2^+/-^ Balb/c mice were fed with methionine and choline deficient diet for 3, 10 and 40 day to induce an early and later-stage of NAFLD, respectively. Fresh fecal samples were collected and followed by 16S rRNA sequencing. HPLC-MRM relative quantification analysis was used to identify changes in hepatic lipid profiles. The expression level of innate immunity-, lipid metabolism- and intestinal permeability-related genes were determined. A spearman’s rank correlation analysis was performed to identify possible correlation between hepatic medium and long-chain fatty acid and gut microbiota in ASPP2-deficiency mice.

**Results:**

Compared with the WT control, ASPP2-deficiency mice developed moderate steatosis at day 10 and severe steatosis at day 40. The levels of hepatic long chain omega-3 fatty acid, eicosapentaenoic (EPA, 20:5 n-3) and docosahexaenoic (DHA, 22:6 n-3), were decreased at day 10 and increased at day 40 in ASPP^+/-^ mice. Fecal microbiota analysis showed significantly increased alpha and beta diversity, as well as the composition of gut microbiota at the phylum, class, order, family, genus, species levels in ASPP2^+/-^ mice. Moreover, ASPP-deficiency mice exhibited impaired intestinal barrier function, reduced expression of genes associated with chemical barrier (REG3B, REG3G, Lysozyme and IAP), and increased expression of innate immune components (TLR4 and TLR2). Furthermore, correlation analysis between gut microbiota and fatty acids revealed that EPA was significantly negatively correlated with Bifidobacterium family.

**Conclusion:**

Our findings suggested that ASPP2-deficiency promotes the progression of NAFLD, alterations in fatty acid metabolism and gut microbiota dysbiosis. The long chain fatty acid EPA was significantly negatively correlated with Bifidobacterial abundance, which is a specific feature of NAFLD in ASPP2-deficiency mice. Totally, the results provide evidence for a mechanism of ASPP2 on dysregulation of fatty acid metabolism and gut microbiota dysbiosis.

## Introduction

Nonalcoholic fatty liver disease (NAFLD), characterized by excessive accumulation of lipids in hepatocytes, has become a major global health problem and leads to a series of pathological changes including simple steatosis, nonalcoholic steatohepatitis, cirrhosis and eventually to hepatocellular carcinoma (1). Several extrahepatic risk factors, such as obesity, insulin resistance, dyslipidaemia, cardiovascular disease, sarcopenia and gut dysbiosis, have been reported to be closely associated with the development of NAFLD (2–6). The microbiota plays an important role in the metabolic system through acting as a “virtual metabolic organ” and forming an axis with a certain number of extraintestinal organs (7). The bidirectional relationship between the gut microbiota and liver, termed the “gut-liver axis”, has been shown to be driven by complex interplay between liver injury, gut microbiota dysbiosis, intestinal barrier dysfunction, homeostasis imbalance of metabolite and dysregulation of the immune system, which contributes to the pathogenesis of NAFLD (8). Studies in animals and humans have also shown that high-fat diet-induced hepatic steatosis can lead to increased intestinal permeability and dysbiosis (9–11). In turn, the gut microbiota has tremendous impact on lipid metabolism in NAFLD patients through producing a variety of metabolites such as short-chain fatty acids (SCFA; acetate, propionate, and butyrate) and secondary bile acids, that cannot be synthesized by mammalian hosts (12). Gut microbiota also induces the generation of mono-unsaturated fatty acid by stearoyl-CoA desaturase 1 and elongation of polyunsaturated fatty acid by fatty acid elongase 5, while acetate from gut microbial degradation serves as precursor of C16 and C18 fatty acids for hepatic synthesis (13). In addition, intestinal macrophages display enhanced antimicrobial activity through butyrate-induced mTOR-dependent LC3-associated cell-clearing systems (14). Gut microbiota has an effect on maintaining the balance of host immune homeostasis by interacting with the innate and adaptive immune system in the intestine. Mao et al. demonstrated that gut microbiota can activate innate cells prior to the development of adaptive immune system in adaptive-lymphocyte-deficient Rag1^-/-^ mice, leading to constitutive activation of group 3 innate lymphoid cells (ILC3s) and intestinal epithelial cells (IECs), which contribute to reduce the expression levels of lipid transporters-related genes and decreased serum lipid concentration (15). Recent studies have shown that probiotics in the intestine have beneficial therapeutic effects on animal models of liver injury, suggesting that gut microbiota is a promising target for treatment of NAFLD (16, 17).

Fatty acids (FAs), existing in the organism in the form of triglycerides, phospholipids and cholesterol esters, are an important component of lipids and the main source of energy for human tissues (18, 19). FAs are classified short-chain fatty acids (≤6 carbons), medium-chain fatty acids (6-12 carbons) and long-chain fatty acids (≥12 carbons) according to their aliphatic tail length (20). Various physiological and biological functions performed by the liver, including lipid homeostasis, signal transduction and endocrine control, depend on the uptake of FAs from the circulation (21). Abnormally elevated levels of FAs in circulation have been closely linked to hepatocellular lipotoxicity, which leads to the early onset of NAFLD (22). The gut microbiota has been reported to be involved in the modulation of lipotoxicity by metabolism of nutrient and other dietary components (23). Dysbiosis of the gut microbiota disturbs the balance of circulating lipid levels and further lead to liver injury (24).

ASPP2, encoded by the tumor protein p53 binding protein 2 (TP53BP2) gene, is a member of the Ankyrin-repeat-SH3-domain and proline-rich region containing protein family (25). As a tumor suppressor, ASPP2 has been shown to inhibit epithelial-mesenchymal transition by regulating epithelial cadherin and β-catenin functions and plays an important role in the tumor microenvironment (26). Moreover, ASPP2 could regulate cell growth and apoptosis by interacting with P53 and its signal-related proteins (27). The p53 pathway has been reported to be involved in the regulation of lipid metabolism through enhancing fatty acid oxidation and inhibiting fatty acid synthesis (28). Our previous study also showed that overexpression of ASPP2 reduced the level of triglyceride and total cholesterol in both the serum and liver using choline deficiency (MCD) diet-induced NAFLD mice model (29, 30). In addition, ASPP2 has also been reported to be involved in autophagy and apoptosis processes (29, 30). All the above findings indicated that the ASPP2 has a potential regulatory role in lipid metabolism that may help prevent liver damage.

To explore the effect of ASPP2 on NAFLD, we established an MCD diet-induced NAFLD model using the ASPP2 globally heterozygous knockout mice in this study. Hepatic metabolomics and microbiota were performed to identify ASPP2-dependent changes in metabolite profiles and the abundance of gut microbiome, respectively. Furthermore, we performed a correlation analysis to reveal the association between hepatic fatty acids and gut microbiota in ASPP2-deficiency mice.

## Materials and methods

### Animals and diets

All animal care and experimental procedures were approved by the Animal Ethics Committee of Capital Medical University (Ethics number: AEEI-2020-167). Balb/C WT mice (Academy of Military Medical Sciences, China) and ASPP2 globally heterozygous knockout mice (ASPP2^+/-^, Gempharmatech, Ltd, China) were maintained according to the guide of laboratory. Mice were fed with a methionine and choline deficient (MCD, Trophic Animal Feed High-tech Co, Ltd, China) diet for 3, 10 and 40 days to induce NAFLD, while a methionine and choline diet supplement (MCS, Trophic Animal Feed High-tech Co, Ltd, China) diet with the addition of sufficient choline bitartrate (2 g/kg) and dl-methionine (3 g/kg)) diet served as normal control. Mice were sacrificed under an anesthesia condition of pentobarbital (50 ml/kg) by intraperitoneal injection. Fresh stool samples were collected and stored at -80°C prior to sacrifice, and serum and tissues such as liver, intestine were collected for further analysis.

### Serum biochemical analysis

The serum aspartate aminotransferase (AST) (Lb20701, Prodia Diagnostics, Germany), alanine aminotransferase (ALT) (Lb20702, Prodia Diagnostics, Germany), total cholesterol (TC) (Lb20101, Prodia Diagnostics, Germany) and triglyceride (TG) (Lb20201, Prodia Diagnostics, Germany) levels were carried out. TC assay kit (Cat# E1026-105, Applygen, China) and TG assay kit (Cat# E1025-105, Applygen, China) were used to measuring the tissue sample following manufacturers’ instructions.

### Liver and intestine histology

Tissue samples such as the liver and intestine were fixed in 10% buffered-neutral formalin. Paraffin-embedded sections were made from Servicebio company (China) according to a standard histological procedure. Hematoxylin and eosin (H&E) staining was performed to evaluate the lipid accumulation in hepatocytes and intestinal barrier dysfunction between groups.

### 16S rRNA sequencing

The CTAB/SDS method was used to extract the total genome DNA from frozen stool samples and DNA was purified by 1% agarose gels. 16S rRNA genes of distinct regions were amplified used specific primer with the barcode and Phusion^®^ High-Fidelity PCR Master Mix (New England Biolabs) following a standard PCR condition (initial denaturation at 98°C for 1 min, followed by 30 cycles of denaturation at 98°C for 10 s, annealing at 50°C for 30 s, elongation at 72°Cfor 30 s and a final elongation at 72°C for 5 min). PCR products were mixed in equidensity ratios and then purified by Gene JETTM Gel Extraction Kit (ThermoFisher Scientific, USA). Libraries were prepared according to manufacturer’s recommendations using the Ion plus Fragment Library Kit (ThermoFisher Scientific, USA). The Qubit@ 2.0 Fluorometer was used to assess the quality of library. Finally, the library was sequenced on an Ion S5TMXL platform and 400bp/600 bp single-end reads, and OTU cluster and species annotation, alpha diversity, beta diversity, function prediction were used to for data analysis. The resulting p-values were corrected for multiple comparisons using the Benjamini–Hochberg correction (False discovery rate (FDR), q-value) for both alpha and beta diversity analyses. A q-value <0.05 was considered statistically significant.

### Lipid extraction

Total lipid was extracted from liver tissues using a modified version of the Bligh and Dyer’s method as described previously (31). Briefly, tissues were homogenized in 750 µL of chloroform: methanol 1:2 (v/v) with 10% deionized water and then incubated at 4°C for 30 min. At the end of the incubation, 350 µL of deionized water and 250 µL of chloroform were added. The samples were then centrifuged and the lower organic phase containing lipids was extracted into a clean tube. Lipid extraction was carried out twice and the lipid extracts were pooled into a single tube and dried in the SpeedVac under OH mode. Samples were stored at -80°C until further analysis.

### Mass spectrometry analyses

Lipids were separated by normal phase (NP)-HPLC using a Phenomenex Luna 3µm-silica column (internal diameter 150 × 2.0 mm) with the following conditions: mobile phase A (chloroform: methanol: ammonium hydroxide, 89.5:10: 0.5) and mobile phase B (chloroform: methanol: ammonium hydroxide: water, 55:39:0.5:5.5). Fatty acids were quantitated using d31-16:0 (Sigma-Aldrich) and d8-20:4 (Cayman Chemicals) as internal standards.

### Gene expression analysis

Total RNA was isolated from the intestine and livers tissues using Trizol reagent (Servicebio, Wuhan, China). cDNA was synthesized by RevertAid First Strand cDNA Synthesis Kit (Thermo, Massachusetts, USA) according to the manufacturer’s recommendation. Quantitative real-time PCR was used to quantify the mRNA levels using ABI 7300 Real-Time PCR system (Applied Biosystems, Foster City, CA, USA) with Faststart Universal SYBR Green Master (Servicebio, Wuhan, China). Primers were synthesized from Biomed Chemicals (Biomed, Beijing, China) and the sequence details are listed in Supporting Information (**Table S1**). Amplifications were performed in the following two steps: 95°C for 10 minutes, to denature DNA and followed by 40 cycles of 95°C for 15 seconds, 60°C for 60 seconds to anneal and extend DNA. Fold change of gene expression level was calculated based on the threshold cycle method (2^-ΔΔCT^).

### Immunoblotting analysis

Liver proteins were extracted from ASPP2^+/-^ and WT mice using tissue lysis buffer (50 mM Hepes, pH 7.5, 150 mM NaCl, 10% Glycerol, 1% Triton X-100, 1.5 mM MgCl_2_, 1 mM EGTA, 10 mM Sodium Pyrophosphate, 100 mM Sodium Fluoride) with a mixture of protease inhibitors including 1 mM PMSF and 10 µg/ml complete protease inhibitor mixture (Roche). The protein concentration was determined by BCA protein assay kit (ThermoFisher Scientific, USA). Immunoblot analysis was preformed following a standard procedure. Briefly, the protein samples went through 10% sodium dodecyl sulfate polyacrylamide gel electrophoresis and then transferred to polyvinylidene fluoride membranes (Millipore, USA), and blocked with 5% non-fat milk. The membranes were incubated with primary antibodies (ASPP2 Rabbit monoclonal antibody (ab181377, Abcam, USA), toll-like receptor 4 (TLR4) Rabbit polyclonal antibody antibody (A0007, Abclonal, China), toll-like receptor 2(TLR2) Rabbit polyclonal antibody antibody (A20608, Abclonal, China), interleukin 1 receptor associated kinase 4(IRAK4) Rabbit polyclonal antibody antibody (A6208, Abclonal, China) and glyceraldehyde-3-phosphate dehydrogenase(GAPDH) Rabbit antibody (#5174, Cell signaling technology, USA)) for overnight at 4°C and anti-rabbit IgG HRP-conjugated secondary antibodies (#7074, Cell signaling technology, China) sequentially. Bands were visualized using the ChemiDocTM MP Imaging System (Bio-Rad, USA) and quantified by image J software (Version 1.51K).

### Statistical analysis

The Data were expressed as mean ± SD and analyzed using Graphpad Prism version 8.3.0 (Graphpad Software, USA). Student t tests were analyzed for differences between two groups, and one-way or two-way analysis of variance (ANOVA) was used for difference among more than two groups as appropriate. The correlation between hepatic key medium and long-chain fatty acid and altered gut microbiota was examined using the Spearman’s rank correlation coefficient. The lipid droplets area of liver was quantified by NIH Image J software (https://imagej.nih.gov/ij/).To detect bacterial taxa with significantly different abundances between the WT and ASPP2^+/-^ groups, Linear discriminant analysis (LDA) coupled with effect size (LEfSe) measurements were used to identify taxa significantly different (biomarkers) among groups, with an LDA score threshold of 4 and P<0.05. P-value of <0.05 was considered statistically significant and p values are shown as *p < 0.05, **p < 0. 01 and ***p < 0. 001.

## Results

### ASPP2-deficiency increases MCD diet-induced hepatic steatosis and liver injury

**Figure 1:**
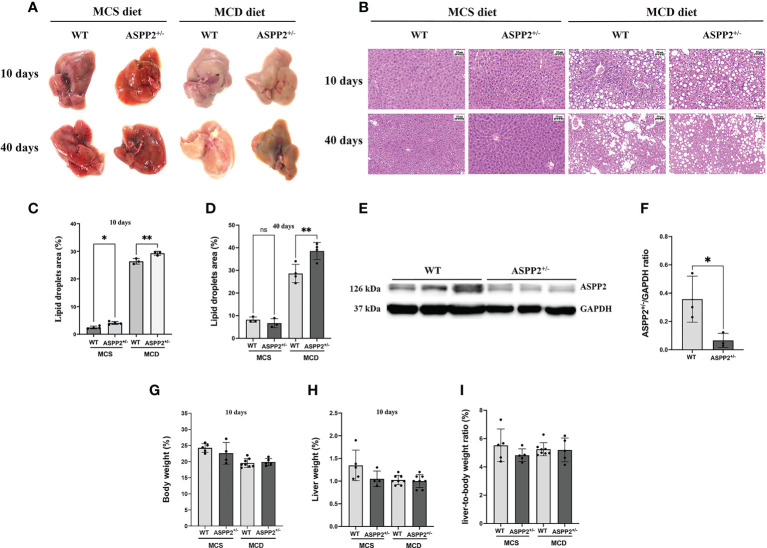
ASPP2 deficiency increased MCD and MCS diet-induced hepatic steatosis. **(A)** Representative macroscopic images of the livers of WT and ASPP2^+/-^ mice induced by MCD or MCS diet. **(B)** H&E staining of liver sections of WT and ASPP^+/-^ mice. Quantification of the mean area of lipid droplets at days 10 **(C)** and 40 **(D)** in WT and ASPP2^+/-^ mice groups. **(E, F)** The expression of ASPP2 protein in the liver of WT and ASPP2^+/-^ mice. The liver weight **(G)**, body weight **(H)**, the liver-to-body weight ratio (%) **(I)** in WT and ASPP2^+/-^ mice at 10 days induced by MCD and MCS diet. WT-MCS group, n =3-5; ASPP2^+/–^MCS group, n =3-4; WT-MCD group, n =3-8; ASPP2^+/-^ -MCD group, n =3-8. *p < 0.05; **p < 0.01. ns, no significance.

**Figure 2:**
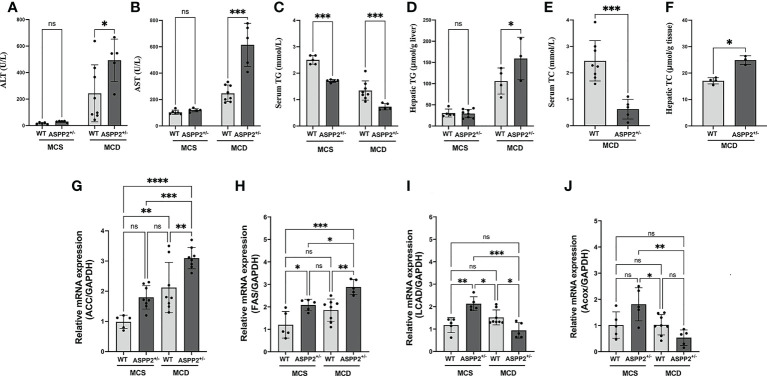
Blood chemistry analysis and expression of genes involving in lipid metabolism of ASPP2 knockout mice fed by MCS or MCD diet. **(A)** The serum ALT, **(B)** serum AST, **(C)** serum TG, **(D)** hepatic TG, **(E)** serum TC and **(F)** hepatic TG were measured using the ELISA kit; The mRNA expression levels of genes associated with fatty acid synthesis **(G, H)** and β-oxidation **(I, J)**. WT-MCS group, n = 5; ASPP2^+/–^MCS group, n = 4; WT-MCD group, n = 8; ASPP2^+/–^MCD group, n =5. *p < 0.05; **p<0.01; ***p<0.001; ****p < 0.0001. ns, no significance.

We treated the Balb/C WT mice and ASPP2 KO Balb/C mice with MCD or MCS diet for 3, 10 and 40 days, respectively. Liver biopsy and serum biochemical analysis was used to access the severity of NAFLD. The results showed that both WT and ASPP2^+/-^ mice had significantly increased lipid droplet accumulation after 10 and 40 days of MCD dietary feeding compared to MCS diet (**Figures 1A–D**), while body weight, liver weight and liver to body weight ratio of each group were not statistically different (**Figures 1G–I**). The expression level of ASPP2 protein in the liver of KO mice was greatly reduced by 87% compared to WT mice (p<0.05) (**Figures 1E, F**). In MCD group, histological analysis revealed that ASPP2^+/-^ mice had significantly higher hepatic lipid deposition than WT mice at 10 and 40 days (p<0.01) (**Figure 1B**). The average area of lipid droplets in ASPP2^+/-^ mice was increased by 10.7% at 40 days compared with 10 days of MCD dietary feeding (**Figures 1C, D**), suggested that ASPP2 deficiency induce moderate and severe steatosis at different time points. On 10 days of MCD dietary feeding, serum ALT, AST, hepatic TG and hepatic TC levels significantly increased in ASPP2^+/-^ mice compared to the WT mice (p<0.05, p<0.01, p<0.05, p<0.05, respectively), while serum TG and TC levels decreased (p<0.001 and p<0.05) (**Figures 2A–F**).

To assess the molecular changes of ASPP2 inhibiting, real-time PCR was performed to determine the gene expression that involved in lipid metabolism and fatty acid oxidation at 10 days of MCD diet induction. The lipogenic genes including ACC (Acetyl-CoA carboxylase) and FAS (fatty acid synthase) mRNA levels were obviously enhanced in the liver of ASPP2^+/-^ mice in MCD group (p<0.05 and p<0.01) compared to the WT mice (**Figures 2G, H**). Meanwhile, expression of long-chain acyl-CoA dehydrogenase (LCAD) and Acyl-CoA oxidase (ACOX) were significantly decreased in ASPP2 deficiency mice treated with MCD (p<0.05), suggested that ASPP2 deficiency promotes fatty acid oxidation disorders (**Figures 2I, J**).

### ASPP2 deficiency reduces the content of long-chain fatty acids in early-stage NAFLD

**Figure 3:**
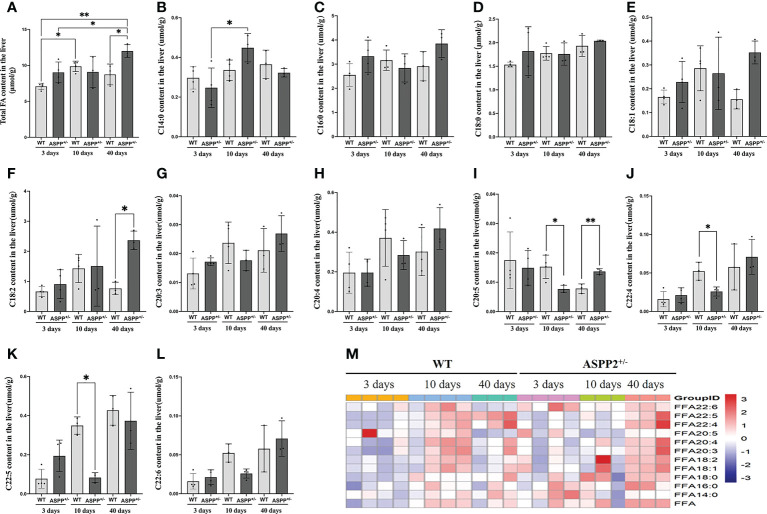
Effect of ASPP2 knockout on FFA metabolism. **(A)** Total fatty acid levels at days 3, 10 and 40 in WT and ASPP2^+/-^ mice groups induced by MCD diet. **(B–M)** The levels of C14:0, C16:0, C:18:0, C18:1, C18:2, C20:3, C20:4, C22:4, C22:5 and C22:6 content at days 3, 10 and 40 in WT and ASPP2^+/-^ mice groups induced by MCD diet. WT-MCD, n = 3-4; ASPP2^+/–^MCD, n =3-4. *p < 0.05; **p<0.01.

To investigate the effect of ASPP2 deficiency on lipid metabolism, we analysis the change in hepatic fatty acid in ASPP2^+/-^ and WT mice induced by MCD diet at different time points using mass spectrometer. WT mice showed significantly increased total fatty acid levels at days 10 and an increased trend at day 40 compared to days 3 (**Figure 3A**). Meanwhile, total fatty acid concentrations were significantly higher in ASPP2 deficiency mice than WT after 40 days of MCD induction (p<0.05), but there was no difference between days 3 and 10 (**Figure 3A**).

The long-chain fatty acid profile has been reported to associate with hepatic steatosis and the development of NAFLD (32). Based on the above, we further analyzed the content of long-chain fatty acids in MCD diet-induced WT and ASPP2^+/-^ mice by lipidomics analyses. WT mice in MCD group showed a gradual upward or increasing trend in the levels of C14:0, C16:0, C:18:0, C18:1, C18:2, C20:3, C20:4, C22:4, C22:5 and C22:6 content between 3 and 10 days (early-stage of NAFLD), but C18:1, C18:2 and C22:6 dropped at 40 days (late-stage of NAFLD) (**Figures 3B-H, J-L**). Meanwhile, the amount of C20:5 gradually decreased over within 3 to 40 days (**Figure 3I**). Unlike WT mice, C18:1, C18:2, C20:4, C22:4 and C22:5 levels in ASPP2 deficiency mice showed a gradual increased between 3-40 days (**Figure 3I**). The amount of C20:5 and C22:5 was significantly reduced in ASPP2^+/-^ mice at 10 days compared to WT mice (p<0.01) but increased at 40 days (**Figures 3I, K, M**), suggested that ASPP2 deficiency may affect long-chain fatty acids metabolism in early-stage of NAFLD.

### ASPP2 deficiency associated with alterations of gut microbiota abundance and diversity

**Figure 4:**
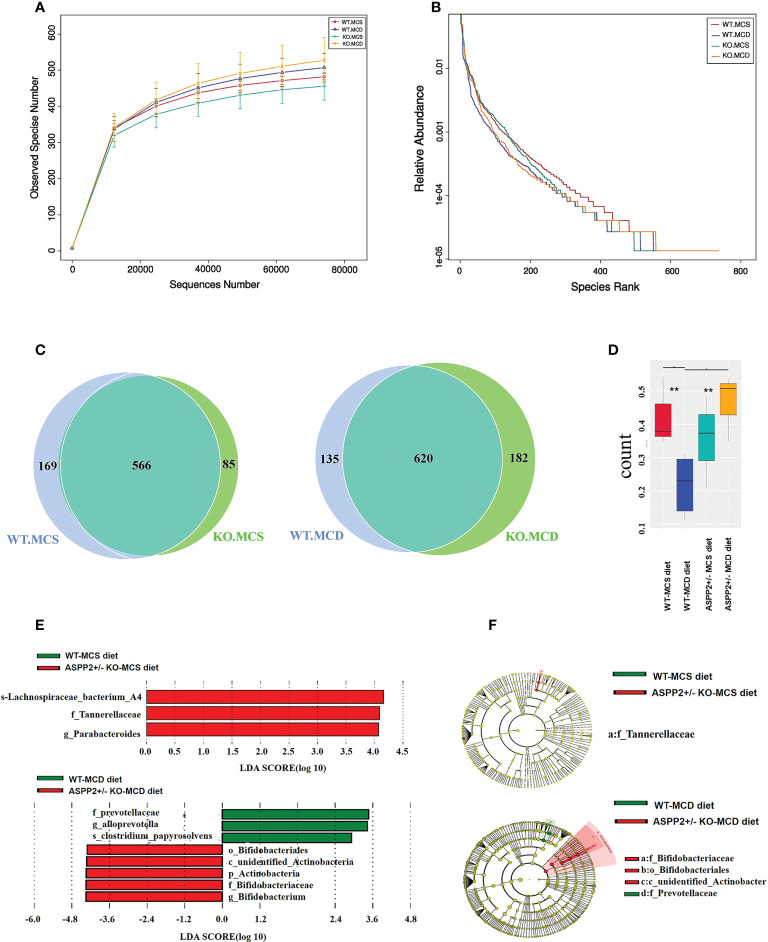
Changes of gut microbiota abundance and diversity induced by MCD or MCS diet for 10 days. **(A, B)** alpha-diversity of gut microbiota based on the OTU data(observer species number and relative abundance. **(C)** Venn diagram (WT-MCS vs ASPP2^+/–^MCS; WT-MCD vs ASPP2^+/–^MCD). **(D)** β-diversity in the indicated groups. **(E)** Histogram represents the linear discriminant analysis (LDA) scores of bacteria with significant differential abundance between WT and ASPP2^+/-^ mice groups induced by MCS or MCD diet identified by different colors. **(F)** Cladogram represents the taxonomic tree of differentially abundant taxa. WT-MCS group, n = 3; ASPP2^+/–^MCS group, n = 3; WT-MCD, n = 4 and ASPP2^+/–^MCD, n = 3. **p<0.01.

ASPP2^+/-^ and WT mice were induced by MCD or MCS diet for 10 days and the stool samples were collected for 16s rRNA microbiota analysis at the end of the treatment. An average of 83,806 reads were measured for each sample through shear filtering of reads, and 79,983 effective data were obtained after quality control (the quality control efficiency is 95.47%). 1033 Operational Taxonomic Units (OTUs) were identified by clustering sequences into OTU. Analysis of alpha diversity has revealed that ASPP2 deficiency increased the number of observer species and relative abundance compared to the WT mice induced by MCD or MCS diet, respectively (**Figures 4A, B**). The Venn graph showed that about 566 OUTs were shared between ASPP2^+/-^ and WT group in MCS diet group, while 620 OUTs were shared in MCD group (**Figure 4C**). Interestingly, we also found that the number of unique taxa in WT mice decreased from 169 in the MCS diet group to 135 in the MCD diet group but increased from 85 to 182 in ASPP2^+/-^ mice, suggesting that ASPP2 deficiency can significantly alter the richness of the gut bacterial community (**Figure 4C**). In addition, beta-diversity boxplots also showed a significant difference in species diversity between the ASPP2^+/-^ and WT group in MCD group (p<0.01), but not in the MCS group (**Figure 4D**).

### ASPP2 deficiency affect the composition of gut microbiota community

**Figure 5:**
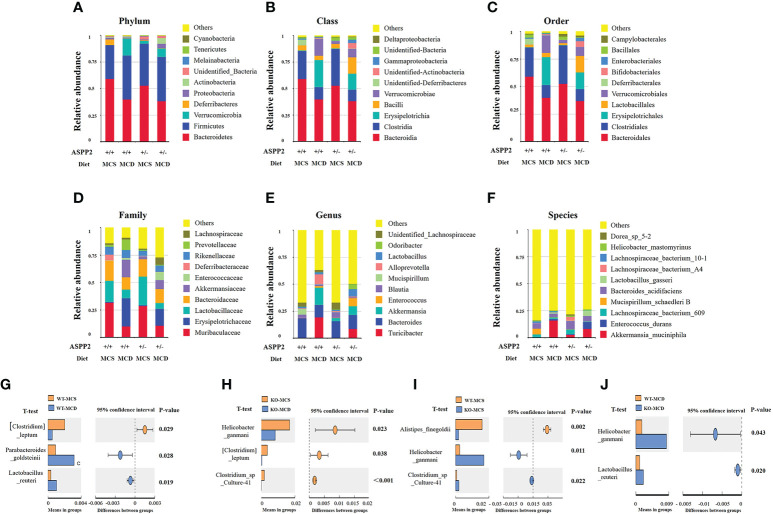
Stacked bar plots depicting phylum **(A)**, class **(B)**, order **(C)**, family **(D)**, genes **(E)** and Species **(F)**-level differences in gut microbiota composition between the ASPP2^+/-^ and WT mice groups with MCS or MCD diet. **(G–J)** Changes in species richness in ASPP2^+/-^ and WT groups of mice induced by MCD and MCS diet for 10 days. Different microbiota detected by T-test.

After MCD diet inducing for 10 days, ASPP2^+/-^ mice exhibited significant difference in the composition of bacterial communities at the phylum/class/order/genus/species level when compared to WT mice. The Firmicutes at the phylum levels were increased in both WT and ASPP2^+/-^ mice of the MCD group compared to the MCS group, but Bacteroidetes phylum levels were decreased. In the MCD group, ASPP2^+/-^ mice also showed an upward trend in Firmicutes/Bacteroidetes ratios relative to WT mice (1.09 vs1.03) (**Figure 5A**).

We next examined the top 10 most abundant bacterial taxonomy. The results showed that the abundance of bacteria of Firmicutes, Bacteroidetes and Verrucomicrobia phylum were the most dominant change between MCS and MCD diet. Compared to the MCS group, enhanced bacterial immunity of Erysipelotrichaceae family, Erysipelotrichales order, Lactobacillales order, Erysipelotrichia class, Turicibacter genus, Verrucomicrobiales order, Verrucomicrobiae class, Akkermansiaceae family, Akkermansia genus were found in MCD group, whereas the composition of Clostridia class, Clostridiales order, Lactobacillaceae family and Blautia genus, Bacteroidia class, Bacteroidales order, Blautia genus and Bacteroides genus were reduced (**Figures 5A–F**).

For MCD group, the bacilli class, Lactobacillales order, Akkermansiaceae family, Enterococcaceae family, Lachnospiraceae family, Lactobacillus genus, Odoribacter genus and Enterococcus genus were increased in ASPP2^+/-^ mice when compared with WT, while Erysipelotrichia class, Verrucomicrobia class, Erysipelotrichales order, Verrucomicrobiales order, Erysipelotrichaceae family, Lactobacillaceae family, Akkermansiaceae family, Prevotellaceae family, Turicibacter genus, Akkermansia genus and Alloprevotella genus were decreased (**Figures 5A–F**).

### The impact of ASPP2 deficiency on gut microbiota at the species level

To determine the effect of ASPP2 on species in the gut microbiota, we further analyzed changes in species richness in different groups of mice induced by MCD or MCS diet for 10 days. WT mice have shown a significantly increased the relative abundance of Parabacteroides_goldsteinii (p<0.05) and Lactobacillus_reuteri (p<0.05) and decreased the abundance of Clostridium leptum (p<0.05) in MCD group when compared to MCS group (**Figure 5G**). Unlike WT mice, the abundance of Helicobacter_ganmani (p<0.05), Clostridium leptum (p<0.05) and Clostridium_sp_Culture-41 (p<0.001) was significantly higher in MCD diet-induced ASPP2^+/-^ mice than the MCS diet (**Figure 5H**).

In MCS group, ASPP2^+/-^ mice have shown a significantly reduced the relative abundance of Helicobacter_ganmani (p<0.05), Clostridium_sp_Culture-41 increased (p<0.05) and Alistipes_finegoldii (p<0.05) compared with WT control (**Figure 5I**). Furthermore, the abundance of Helicobacter_ganmani (p<0.05) and Lactobacillus_reuteri (p<0.05) were significantly increased in the ASPP2^+/-^ of MCD group compared to WT control (**Figure 5J**).

### ASPP2 deficiency impairs intestinal barrier function

**Figure 6:**
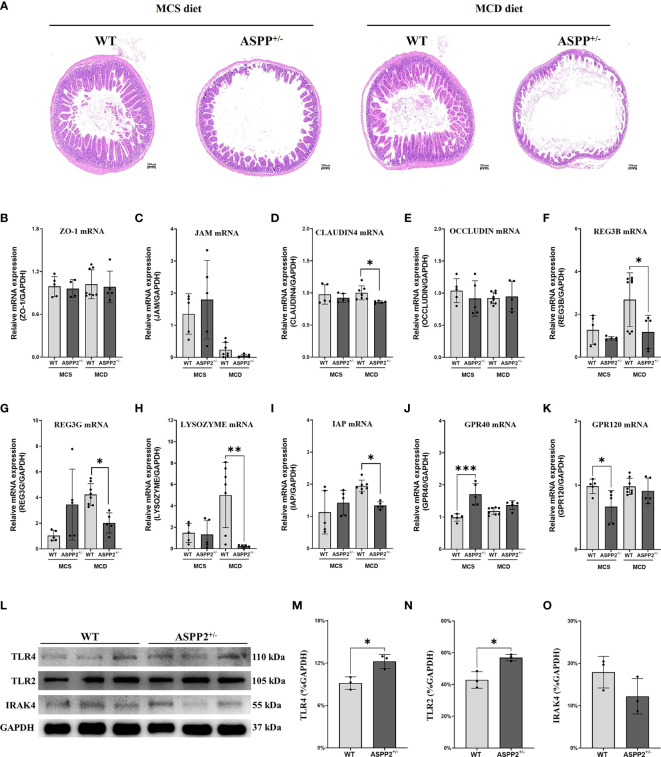
MCD and MCS diet for 10 days deteriorated intestinal barrier function and elevated the level of intestinal fatty acid receptors in ASPP2^+/-^ mice group. **(A)** Representative intestine H&E staining sections of WT and ASPP^+/-^ mice induced by MCD or MCS diet for 10 days. **(B–F)** the mRNA expression levels of intestinal barrier function were measured in the indicated groups. **(G–K)** The mRNA expression levels of genes associated with intestinal fatty acid receptor. **(L–O)** The expression of TLR4, TLR2 and IRAK4 protein in the liver of WT and ASPP2^+/-^ mice. WT-MCS group, n=3-5; ASPP2^+/–^MCS group, n=3-5; WT-MCD groups, n = 3-8; ASPP2^+/–^MCD groups, n = 3-8. *p < 0.05; **p<0.01; ***p<0.001.

Enhanced intestinal epithelial permeability has been reported to facilitate the transport of multiple enteric pathogen-associated microbial products which involves the progression of NAFLD (33). Accordingly, we further analyzed the impact of ASPP2-deficiency on the intestinal barrier by histopathology of small intestine. In MCS group, there were not significant differences in the intestinal villi and epithelium between WT and ASPP2 deficiency mice at 10 days. In contrast, ASPP2-deficiency mice had a reduced number of intestinal villi and blunt villi, thinner epithelium, and irregular epithelial cell arrangement at 10 days in MCD group compared to WT mice (**Figure 6A**).

Furthermore, the gene expression of claudin-4, a marker of epithelium integrity and gut mechanical barrier, was significantly down-regulated in ASPP2-deficiency mice compared with WT mice, whereas other markers such as zonula occludens-1 (ZO-1), Occludin and junctional adhesion molecule (JAM) had no difference between the two groups (**Figures 6B–E**). For the chemical barriers, we found that regenerating islet-derived 3 beta (REG3B), regenerating family member 3 gamma (REG3G), LYSOZYMES and intestinal alkaline phosphatase (IAP) gene expression were significantly decreased in ASPP2^+/-^ mice compared to WT mice, suggesting that ASPP2 deficiency impairs intestinal barrier function (**Figures 6F–I**). In addition, there were no difference of the expression of major fatty acid receptor G-protein-coupled receptor 120 (GPR120) and GPR40 levels in the intestine between WT and ASPP2^+/-^ mice induced by MCD diet for 10 days (**Figures 6J, K**).

In addition, we further found that the expression of innate immune components TLR2 and TLR4 was significantly upregulated in the liver of ASPP2-deficient mice compared with WT mice, while the protein level of IRAK4 was unchanged (**Figures 6L-O**), suggesting that ASPP2 may be involved in hepatic immune regulation.

### Correlations between gut microbiota and hepatic fatty acid

We further performed a Spearman’s rank correlation analysis between hepatic key ASPP2-related medium and long-chain fatty acid and altered gut microbiota in ASPP2-deficiency mice induced by MCD diet for 10 days (**Table 1**). The Actinobacteria phylum has been shown to be significantly negatively correlated with FA 20:5 (**Figure S2**). Interestingly, the Bifidobacteriaceae family and Bifidobacterium genus, which belongs to the Actinobacteria phylum and observed in the LEfSe analysis, were also significantly negative-correlated with FA 20:5 level (**Figures 4E, F**, **S2**). In addition, Fibrosis-related Ruminococcaceae family was negatively correlated with FA 20:4, FA 22:4 and FA 22:5, while the Planococcaceae family and Nocardiaceae family were also negatively correlated to FA 20:4 and FA 22:6 (**Figure S3**). However, the results suggested that enhanced bacterial immunity of Bifidobacteriaceae at family and genus level may contribute to the reduction of FA 20:5 levels in MCD diet induced-ASPP2-deficiency mice (**Figure S4**).

**Table 1:** Correlations between different gut microbiota and key hepatic FFAs.

	*Phylu (negative)*	*Family (positive)*	*Family (negative)*	*genus (positive)*	*genus (negative)*
***Total FA***		Muribaculacea*	unidentified_Bacteroidales* Planococcaceae* Nocardiaceae*	Alistipes*	unidentified_Muribaculacea* Sporosarcina* Rhodococcus* Fermentimonas* Agathobacter*
***FA 14:0***			unidentified_Flavobacteriales* Others* Atopobiaceae* Anaeroplasmataceae*	unidentified_Lachnospiraceae* Bacillus*	Cloacibacterium* Anaeroplasma* Agromyces*
***FA 16:0***		Corynebacteriaceae*	Tannerellaceae* Ktedonobacteraceae* Aerococcaceae*	Fournierella* Aneurinibacillus*	Parabacteroides* Aerococcus*
***FA 18:0***			Sphingobacteriaceae* Rhodothermaceae * Porphyromonadacea* Micrococcaceae* Burkholderiaceae*	Intestinimonas*	unidentified_Erysipelotrichaceae* unidentified_Enterobacteriaceae* Salinibacter* Psychrobacter* Porphyromonas* Parasutterella* Paenibacillus* Dialister* Anaerostipes*
***FA 18:1***				Erysipelatoclostridium* Alistipes*	Caproiciproducens*
***FA 18:2***				Holdemanella* Alistipes*	unidentified_Muribaculacea* Caproiciproducens*
***FA 20:3***					Roseburia* Harryflintia* Caproiciproducens*
**FA 20:4**			unidentified_Bacteroidales* Ruminococcaceae* Planococcaceae* Nocardiaceae*	Butyricimonas*	unidentified_Muribaculacea* Sporosarcina* Roseburia* Rhodococcus* Harryflintia* Fermentimonas*
**FA 20:5**	Actinobacteria(n) *	Rhizobiaceae * Defluviitaleaceae*	Eggerthellaceae* Bifidobacteriaceae*	Ramlibacter* Peptococcus* Mesorhizobium* Leuconostoc* Bilophila*	unidentified_Muribaculacea* Rheinheimera* Phascolarctobacterium* Enterorhabdus* Bifidobacterium*
**FA 22:4**			Ruminococcaceae*	Butyricimonas* Bacillus*	unidentified_Clostridiales* Phascolarctobacterium*
**FA 22:5**		Erysipelotrichaceae* Bacillacea*	Ruminococcaceae*	Bacillus*	unidentified_Clostridiales* Harryflintia*
**FA 22:6**			unidentified_Bacteroidales* Planococcaceae* Nocardiaceae*	Butyricimonas*	unidentified_Muribaculacea* Sporosarcina* Roseburia* Rhodococcus* Fermentimonas*

*p < 0.05.

## Discussion

ASPP2 has been reported to be involved in hepatic lipid metabolism, and its deficiency promotes the development of NAFLD, but the underlying mechanism remains unclear (29). In the present study, we investigated the effect of ASPP2 on gut microbiota and hepatic fatty acid homeostasis in early and later-stage of NAFLD by multi-omics analysis using MCD-induced ASPP2-deficient mice. Compared with WT mice, we observed for the first time that ASPP2-deficient caused (i) moderate steatosis in early-stage of NAFLD and severe liver damage in later-stage of NAFLD, respectively; (ii) significantly alterations in hepatic long chain fatty acid (mainly omega-3 PUFAs) metabolism and gut microbiota composition in the early-stage of NAFLD; (iii) a significant correlation between hepatic FA 20:5 and bifidobacteria family and genus. In addition, dysregulation of several immune-related gut microbiota and metabolites reflects the impact of ASPP2 on host immune status, and its absence leads to an imbalance between innate and adaptive immune system.

As a pleiotropic regulator, ASPP2 has been shown to play a crucial role in cell apoptosis, growth, and differentiation by regulation of autophagy and apoptosis (34). Attenuation of the ASPP2 expression levels have also been found in various human cancers, such as breast cancer, gallbladder cancer, pancreatic cancer, gestational choriocarcinoma and hepatocellular carcinoma (35–39). In previous study, we demonstrated that overexpression of ASPP2 significantly inhibited autophagy, reduced hepatic TG content and lipid droplets in a 10-day MCD diet-induced mouse model, which contributed to the improvement of liver injury (29). More importantly, we found that the expression level of ASPP2 was significantly lower in patients with NAFLD compared to normal subjects, suggesting that ASPP2 is a key factor in the progression of NAFLD and has emerged as a potential novel target for the treatment of NAFLD (29). Therefore, we conducted this study to further elucidate the role and potential molecular mechanisms of ASPP2 in hepatic lipid metabolism and the gut-liver axis using MCD-induced ASPP2**^+/-^** mice.

NAFLD arises from an imbalance in lipid metabolism, leading to the accumulation of TG-rich droplets in the liver, and dysregulation of fatty acids uptake and synthesis, which further promotes the occurrence of lipotoxicity, oxidative stress, inflammation fibrosis and severe liver damage (40, 41). Abnormalities in hepatic fatty acid metabolism are closely associated with the occurrence and progression of NAFLD, including simple hepatic steatosis, nonalcoholic steatohepatitis (NASH), fibrosis, cirrhosis and even HCC (41, 42). Based on the above, we analyzed the effect of ASPP2 deficiency on fatty acid metabolism. The results showed that a decrease in in serum TG levels and an increase in hepatic TG levels in MCD diet-induced ASPP2^+/-^ mice for 10 days compared to WT. In addition, ASPP2-deficient mice exhibited significantly reduced levels of the major long-chain fatty acid C20:5 n-3 (eicosapentaenoic acid, EPA) and C22:6 n-3 (docosahexaenoic acid, DHA) in liver. EPA and DHA are the major components of omega-3 polyunsaturated fatty acids (PUFA, n-3 fatty acids) derived from dietary intake (43, 44) or synthesized from its major precursor α-linolenic acid (omega-3 C18:2 n-3, ALA), which is converted to DHA or EPA by desaturases and elongases (13, 14). Treatment with omega-3 PUFAs in NAFLD patients has also been shown to have beneficial effects on hepatic lipid accumulation, oxidative stress, inflammation and fibrosis (45, 46). Lower intake of omega-3 PUFAs has been reported as a risk factor for the progression of NAFLD (44, 47). However, our study revealed for the first time that ASPP2 has specific effect on the composition of omega-3 PUFAs despite no change in total fatty acid content, a key mechanism by which ASPP2 triggers the initiation of NAFLD.

ASPP2 plays an important regulatory role in apoptosis, autophagy and cell growth by interacting with several proteins including P53, Bcl-2, NF-κB, protein phosphatase-1, Yes-associated protein-1, APCL and HCV core protein (48). Autophagy has also been shown to be closely associated with the synthesis of long chain fatty acids (49, 50). The enhanced autophagy activity can promote the conversion of long-chain acyl-CoA molecule to acetyl-CoA molecule by increasing fatty acid beta-oxidation, furthermore, leading to the breakdown of long chain fatty acids (50). Overexpression of ASPP2 significantly reduced the activity of autophagy in both HepG2 cells and BALB/c mice, while ASPP2 silencing induced autophagic flux through increasing the conversion of LC3I to LC3II (29, 51). Thus, the effect of APSS2 on long-chain fatty acids that we found in the present study is likely due to the activation of autophagy-related lipid metabolic pathways in ASPP2^+/-^ mice.

Imbalance in hepatic lipid metabolism has been shown to be highly associated with dysregulated gut microbiota in NAFLD through involvement in bacterial metabolic processes that use lipids as substrates, and exhibit toxic effects on bacterial growth (13). Several studies have shown broad differences in gut microbiota profile during the progressive stages of NAFLD, including simple steatosis, NASH, fibrosis and cirrhosis (52). In turn, changes in the microbiota can further influence the development of NAFLD by modulating metabolites produced by the gut microbiota (e.g., short-chain fatty acids, secondary bile acids, etc.), pro-inflammatory factors (e.g., lipopolysaccharides) and intestinal permeability and immunity (53). To elucidate the effects of changes in omega-3 PUFAs levels driven by ASPP2-deficiency on the gut microbiota, we first examined the expression levels of the major long-chain fatty acid receptors GPR40 and GPR120 in the small intestine. Both GPR40 and GPR120, members of the G-protein-coupled receptor family, are activated by their endogenous ligands including medium- and long-chain free fatty acids, and exhibit beneficial effect on metabolic and inflammatory diseases (54, 55). We did not find any difference in the expression of these two receptors in MCD-induced ASPP2+/- and WT mice (**Figures 6J, K**). As an important member of the omega-3 PUFAs, α-linolenic acid (ALA) can be converted to DHA or EPA and has a higher content than other omega-3 PUFAs (56). Thus, the unaltered expression levels of GRP40 and GRP120 in ASPP2^+/-^ and WT mice should be influenced by other unchanged medium- and long-chain free fatty acids such as ALA in current study.

Next, we further analyzed the composition and abundance of gut microbial communities in ASPP2^+/-^ and WT mice induced by MCD. At the top 10 most abundant genus, we found an increased abundance of Lactobacillus, Odoribacter, Enterococcus and a decreased abundance of Turicibacter, Akkermansia, Alloprevotella in ASPP2^+/-^ mice compared with WT. Lactobacillus genus, including Lactobacillus acidophilus, Lactobacillus fermentum and Lactobacillus paracasei species, have been found to be significantly less abundant in patients with NAFLD compared to controls and to improve liver damage by lowering cholesterol (57). Unlike the above studies, we found that high abundance of Lactobacillus (A 3.7-fold increase compared to WT mice) in ASPP2-deficient mice was mainly due to the increased Lactobacillus gasseri species (A 4.6-fold increase compared to WT mice). Lactobacillus gasseri has been reported to participate in the process of bile acid deconjugation and suppress lipid reabsorption in the intestine (58, 59). Zeng et al. found a 6900-fold increase of the amount of Lactobacillus gasseri and/or Lactobacillus taiwanensis DNA in mice fed with a high fat containing 45% energy diet compared to mice fed a low fat contain 10% energy diet and was positively correlated with NAFLD progression (60). Except to Lactobacillus, Enterococcus genus were involved in antibiotic resistance and exacerbated the progression of alcoholic liver disease by increasing toll-like receptor 2-mediated sIL-1β secretion on Kupffer cells, while odoribacter genus were also highly associated with NAFLD phenotype in mice induced by high fat diet or high-fructose diet compared to control (61, 62). In addition, decreased abundance of Turicibacter, Akkermansia and Alloprevotella has been reported to be positive associated with the progression of NAFLD (63–65). Consistent with the above-mentioned studies, our findings revealed for the first time the profile of the gut microbiota associated with ASPP2-deficiency, which are involved in promoting the progression of NAFLD.

In addition, decreased abundant of Turicibacter, Akkermansia and Alloprevotella have also been reported to be positive associated with the progression of NAFLD (63–65).

Omega-3 PUFAs have been demonstrated to participate in maintaining gut homeostasis and regulating gut immunity (66). A randomized trial performed by Watson et al. on healthy volunteers over 50 found that an intervention with omega-3 PUFAs (EPA and DHA) significantly increased the abundance of Bifidobacterium, Oscillospira, Lachnospira, and decreased the abundance of Coprococcus and Faecalibacterium (67). Moreover, mouse model supplemented with different doses of omega-3 PUFAs (EPA and DHA) also demonstrated significantly higher abundances of Clostridiales, Lactobacillus, and Bifidobacterium in the 60-mg group compared with 30 and 90 mg group. Among these bacteria, an increase in the abundance of Bifidobacterium was frequently observed to be positively correlated with the supplement of omega-3 PUFAs.

Bifidobacteria is derived from the normal human intestines and have been shown to be beneficial for metabolic syndrome such as obesity, diabetes and NAFLD (68, 69). Million et al. reported that obese individuals had reduced levels of bifidobacteria in the gut microbiota community compared to normal subjects (70). Four bifidobacterial species L66-5, M13-4, FS3-1-1-2 and L75-4 have been proved to promote the reduction of TG levels and hepatic lipid deposition in NAFLD (71). Administration of Bifidobacterium in mice caused increased concentrations of EPA and DHA in adipose tissue (72). Importantly, intake of bifidobacteria could result in consistent improvements in NAFLD (73). Interesting, our results demonstrate that ASPP2-deficiency leads to increased abundance of bifidobacteria and decreased EPA levels in ASPP2^+/-^ mice induced by MCD diet for 10 days. An inverse relationship between bifidobacterial abundance and EPA were found by Spearman’s rank correlation analysis in early stage of NAFLD. Compared with other studies, the different observed in this study may be due to the mechanism by which ASPP2 regulates autophagy. Bifidobacterium has been reported to be involved in the maintaining intestine barrier function by promoting the formation of the Atg12–Atg5–Atg16 complex and initiating autophagy activation in intestinal epithelial cells (74). ASPP2- deficiency enhanced the activity of autophagy, which may further feedback regulate the abundance of Bifidobacterium in the small intestine.

Furthermore, gut microbiota has ability to influence the function of host immunity by interacting with the innate and adaptive immune system in the intestine. Lipopolysaccharide (LPS), a product of the gut microbiome and considered an important molecular marker of microbial invaders, is a major endotoxin that can be recognized by innate immune system in pathogen-associated molecular patterns (PAMPs) (75). As pattern recognition receptors (PRR), Toll like receptor (TLR) 4 binds to LPS and plays an essential role in the activation of innate immunity, while TLR2 also has significant innate immunomodulatory effect by forming a heterodimer with TLR1 or TLR6 and subsequent recognizing its ligand, including triacylglycerol molecules, proteins and polysaccharides (76, 77). The activation of both TLR4 and TLR2 signaling has been reported to strongly contribute to the enhancement of the innate immune systems and to the progression of NAFLD (78, 79). In the present study, we also found that TLR2 and TLR4 protein expression levels were significantly elevated in MCD-induced ASPP2^+/-^ mice compared to WT controls, implying that the presence of high levels of circulating LPS produced from the gut microflora might contribute to the activation of innate immune system.

In addition, the gut microbiota has also been reported to promote the evolution and specific memory of the adaptive immune system through bacterial components and their active metabolites. Short-chain fatty acids (SCFAs), produced mainly by Alloprevotella, Flavonifractor and Oscillibacter in the colon, are involved in the epigenetic regulation of B-cell differentiation by promoting the production of IgA and IgG isoforms (80). Akkermansia muciniphilah species have been shown to enhance the intestinal adaptive immune response in mice by inducing immunoglobulin G1 (IgG1) antibodies and antigen-specific T cell responses, while participating in shaping the development of the adaptive immune system (81). Moreover, omega-3 PUFAs have a function to induce differentiation of T cells into Tregs, and to specifically increase the amount of IgM produced by B cells without altering the levels of IgA, IgG, or IgD (82). In contrast, our results showed that there had significantly lower levels of both EPA and DHA in MCD-induced ASPP2^+/-^ mice, accompanied by a markedly decreasing in the abundance of Alloprevotella and Akkermansia genus, suggesting that ASPP2-deficiency may suppress adaptive immune responses.

One possible explanation for the imbalance between innate and adaptive immunity in ASPP2-deficiency mice is that ASPP2 and its associated gut flora may play a key regulatory role in the adaptive immune system. Mao et al. (15), demonstrated that gut microbiota, such as segmented filamentous bacteria (SFB), can activate innate cells prior to the development of adaptive immune system, further leading to activation of innate lymphoid cells (ILCs) and STAT3 signaling in intestinal epithelial cells (IECs) using adaptive-lymphocyte-deficient Rag1^-/-^ mice, but these signals will be suppressed when adaptive immunity matures (15). An increasing abundant of total bacteria, SFB, bacteroides and Lactobacillaceae in the small intestine also be found positive related to the constitutively activated of group 3 innate lymphoid cells (ILC3s) and IECs in Rag1^-/-^ mice. Furthermore, persistent activation status of ILC3s results in the lower expression levels of lipid transporters-related genes and reduce serum lipid concentration such as triglycerides and free fatty acids (15). Similar to this study, we also found increased abundance of Lactobacillaceae family (3.7 fold increase compared to WT) and Lactobacillus genus (3.7 fold increase compared to WT) despite no significant alteration in SFB, as well as decreased serum triglycerides and hepatic long chain fatty acids levels in the early-stage of NAFLD through an MCD-induced ASPP2-deficient mouse. These findings suggest that ASPP2 can promote adaptive immune maturation through alterations in the gut microbiota, which may be an important mechanism by which ASPP2 deficiency leads to disorders of fatty acid metabolism and the onset of NAFLD. However, the function of ASPP2 in immune regulation needs further investigation.

Our study has some limitations. (i) The present study is the first to explore changes in the gut microbiota profiles associated with ASPP2 deficiency in MCD-induced mice at the level of class (Bacilli and Erysipelotrichia), order (Lactobacillales, Erysipelotrichales and Verrucomicrobiales), family (e.g., Akkermansiaceae, Lachnospiraceae, Erysipelotrichaceae, Lactobacillaceae, etc.), genus (Lactobacillus, Odoribacter, Enterococcus, Turicibacter, Akkermansia and Alloprevotella) and species (Helicobacter_ganmani and Lactobacillus_reuteri). However, there is still a lack of evidence to support these results in patients with NAFLD. (ii) We only observed an association between gut microbiota and fatty acid by a method of statistical analysis, not a causal correlation. Therefore, the causal relationship between gut microbiota and fatty acids in ASPP2-deficient mice has not been described. (iii) There is still a lack of critical data to demonstrate the effect of ASPP2 on the immune system.

In conclusion, our study found that MCD diet-induced ASPP2-deficient mice developed moderate steatosis at 10 days and severe steatosis at 40 days, accompanied by significantly lower levels of hepatic EPA and DHA, and the gut microbiota profile in the early-stage of NAFLD. To our knowledge, this is the first report of the gut microbiota profile associated with ASPP2-deficiency. Furthermore, we found a negative correlation between hepatic FA 20:5 and Bifidobacteria family and genus, which is a specific feature of ASPP2-deficiency-induced NAFLD. Given that the characteristics of the gut microbiota population would serve as the most useful diagnostic biomarkers for NAFLD, as well as the low expression of ASPP2 has been demonstrated in NAFLD patients, our results not only provide evidence for a mechanism of ASPP2 on dysregulation of fatty acid metabolism and gut microbiota dysbiosis, but also demonstrate that ASPP2 is an important potential target for the treatment of NAFLD.

## Data availability statement

The original contributions presented in the study are included in the article/**Supplementary Materials**. Further inquiries can be directed to the corresponding authors.

## Ethics statement

The animal study was reviewed and approved by Animal Ethics Committee of Capital Medical University.

## Author contributions

FX, YZ, Q-HM, YW, and DC conceived and designed the experiments. FX, BK, and M-YC performed the experiments. XL, JW, and JD contributed to reagents and materials. FX and H-FX analyzed the data. FX and YZ wrote the manuscript. All authors contributed to the article and approved the submitted version.

## Funding

This work was supported by the Capital’s Funds for Health Improvement and Research (2018–1–1151); The National Natural Science Foundation of China (81672026, 82070627); The National Science and Technology Major Project of China (2018ZX10302205-005, 2017YFC0211602); Foundation of Beijing Institute of Hepatology (BJIH-01506; BJIH-01610; BJIH-01706; BJIH-2018-3-6); You’ an Foundation of Liver Disease and AIDS, China (BJYAH2016YN02); Beijing Municipal Natural Science Foundation (7192085, 7192084, 7222090); The Ministry of Science and Technology of the People’s Republic of China special project for the prevention and treatment of major infectious diseases such as AIDS and viral hepatitis (2018ZX10302206); The Capital Health Research and Development of Special (No.2020-2-1152); The Beijing Municipal Institute of Public Medical Research Development and Reform Pilot Project (2021-10); The Research Fund of Beijing Institute of Hepatology (Y-2021-6).

## Conflict of interest

The authors declare that the research was conducted in the absence of any commercial or financial relationships that could be construed as a potential conflict of interest.

## Publisher’s note

All claims expressed in this article are solely those of the authors and do not necessarily represent those of their affiliated organizations, or those of the publisher, the editors and the reviewers. Any product that may be evaluated in this article, or claim that may be made by its manufacturer, is not guaranteed or endorsed by the publisher.
