# Supplementary material for: Dysregulated hepatic lipid metabolism and gut microbiota associated with early-stage NAFLD in ASPP2-deficiency mice
Source: Front Immunol. 2022 Nov 18;13:974872. doi: 10.3389/fimmu.2022.974872 (PMC9716097; doi:10.3389/fimmu.2022.974872)
Supplement: Supplementary file 1 [file DataSheet_1.docx]

**A B**


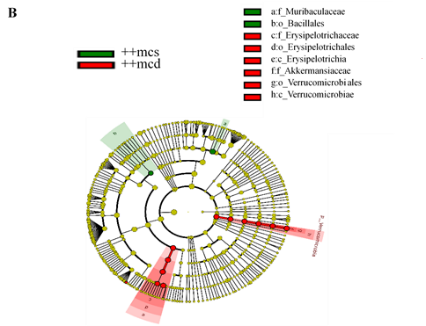

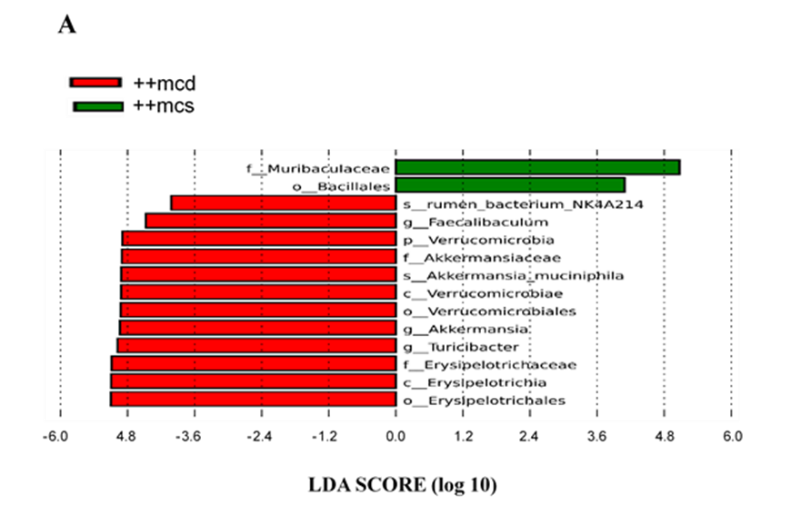


**Supplementary Figure 1. (A)** Histogram represents the linear discriminant analysis (LDA) scores of bacteria with significant differential abundance in WT mice groups induced by MCS and MCD diet identified by different colors. **(B)** Cladogram represents the taxonomic tree of differentially abundant taxa. WT-MCS group, n = 3; WT-MCD group, n = 4.


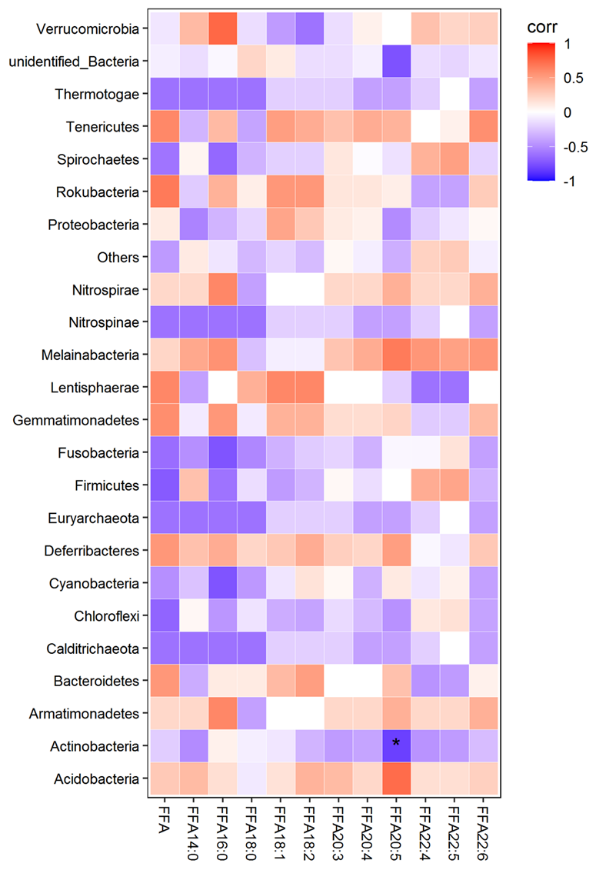


**Supplementary Figure 2.** **Correlation heatmap analysis between the intestinal microbiota and FAs at the phylum level**. Red represents a positive correlation, and blue represents a negative correlation. Asterisks indicate significance (*p < 0.05).


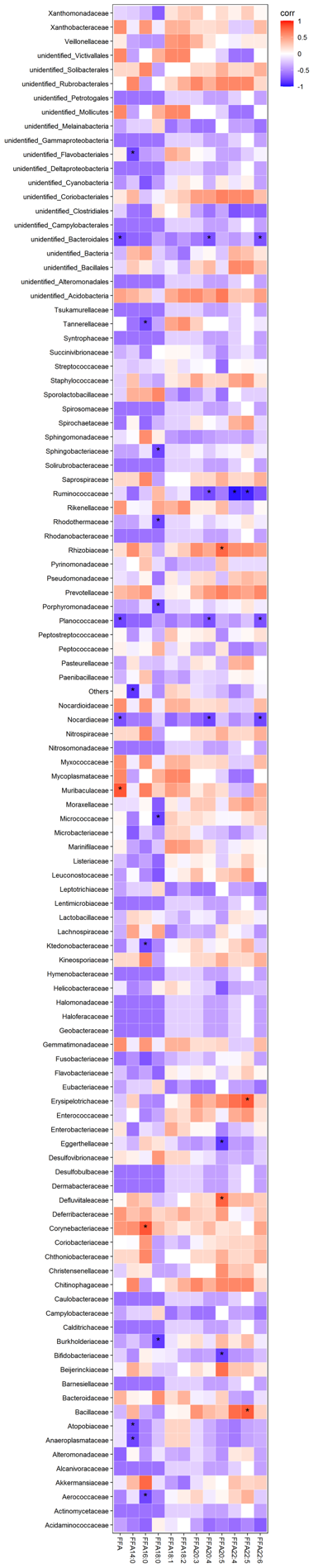


**Supplementary Figure 3.** Correlation heatmap analysis between the intestinal microbiota and FAs at the family level. Red represents a positive correlation, and blue represents a negative correlation. Asterisks indicate significance (*p < 0.05).


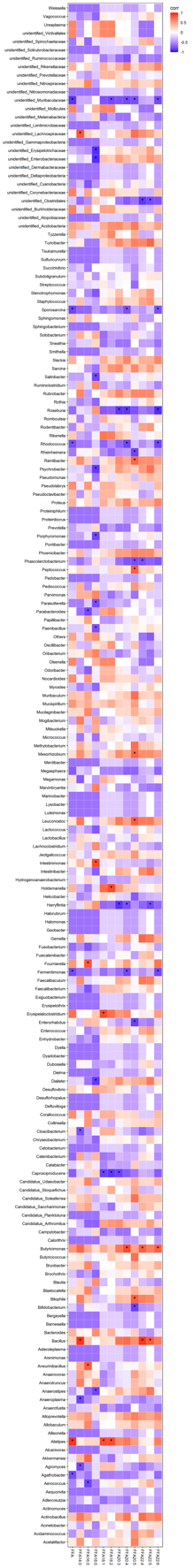


**Supplementary Figure 4.** Correlation heatmap analysis between the intestinal microbiota and FAs at the genus level. Red represents a positive correlation, and blue represents a negative correlation. Asterisks indicate significance (*p < 0.05).

**Supplementary Table 1. ﻿The oligonucleotide primer list.**

| **Gene** | **Forward primer**  (5' −> 3') | **Reverse primer**  (5' −> 3') |
| --- | --- | --- |
| **GAPDH** | CCTCGTCCCGTAGACAAAATG | TGAGGTCAATGAAGGGGTCGT |
| **ACC** | ATGTCTGGCTTGCACCTAGTA | CCCCAAAGCGAGTAACAAATTCT |
| **FAS** | AGATTGTGTGATGAAGGACATGG | TGTTGCTGGTGAGTGTGCATT |
| **LYSOZYME** | CTGGGACTCCTCCTGCTTTCTG | CTTCGGTCTCCACGGTTGTAGT |
| **REG3B** | GCGCTGAGGCTTCATTCTTGT | TGTTACTCCATTCCCATCCACC |
| **IAP** | AAGCAGTGGCTCATCGTACC | GAATATCGCCACGTCCTCCC |
| **GPR120** | CTGTGCAGGAATGAGTGGAAG | CTGATGGAGGGTACTGGAAATG |
| **GPR40** | TTTCATAAACCCGGACCTAGGA | CCAGTGACCAGTGGGTTGAGT |
| **ACOX** | GACTCCAGCCCAGCAATAAA | CAGCCCTCTCACTCCAAGAC |
| **LCAD** | GGATTCCAGGATGTAGGCAG | GGTACATGTGGGAGTACCCG |
| **ZO-1** | GGGAAAACCCGAAACTGATG | GCTGTACTGTGAGGGCAACG |
| **JAM** | CAAGGCAAGGGTTCGGTGTA | GCTGTACTGTGAGGGCAACG |
| **OCCLUDIN** | CCCAGGCTTCTGGATCTATGT | TCCATCTTTCTTCGGGTTTTCA |
| **CLAUDIN4** | TGATTATGGTGCCCGTGTCC | CGAGTAGGGCTTGTCGTTGC |
| **REG3G** | AAGCTTCCTTCCTGTCCTCC | TCCACCTCTGTTGGGTTCAT |
| **IAP** | TCCCAGTATGTTTGGAATCGTG | CGAACATCACAGCCTAGTCAG |

**Abbreviations**

| ASPP2 | apoptosis‐stimulating protein two of p53 |
| --- | --- |
| NAFLD | non-alcoholic fatty liver disease |
| NASH | non-alcoholic steatohepatitis |
| WT | wild type |
| KO | knock out |
| MCS | methionine and choline diet supplement /a control diet |
| MCD | methionine and choline deficient diet |
| PUFA | polyunsaturated fatty acids |
| ALT | alanine aminotransferase |
| AST | aspartate aminotransferase |
| TG | triglycerides |
| TC | total cholesterol |
| ACC | acetyl-CoA synthase |
| FAS | fatty acid synthase |
| LCAD | long-chain acyl-CoA dehydrogenase |
| ACOX1 | peroxisomal acyl‐CoA oxidase 1 |
| FA | fatty acid |
| EPA | eicosapentaenoic acid |
| DHA | docosahexaenoic acid |
| LCFA | long chain fatty acid |
| ZO-1 | zonula occludens‐1 |
| JAM | junctional adhesion molecule |
| IAP | intestinal alkaline phosphatase |
| FFAR1/ GPR40 | free fatty acid receptor1/ g protein coupled receptor40 |
| FFA4 /GPR120 | free fatty acid receptor4/ g protein coupled receptor120 |
| TLR2 | Toll-like receptor 2 |
| TLR4 | Toll-like receptor 4 |
| IRAK4 | interleukin 1 receptor associated kinase 4 |
| H&E | hematoxylin and eosin |
